# System Analysis of Adaptor-Related Protein Complex 1 Subunit Mu 2 (AP1M2) on Malignant Tumors: A Pan-Cancer Analysis

**DOI:** 10.1155/2022/7945077

**Published:** 2022-02-10

**Authors:** Yuanxue Yi, Qiufang Zhang, Youfeng Shen, Yueyi Gao, Xiaohong Fan, Sini Chen, Xin Ye, Jian Xu

**Affiliations:** ^1^Chongqing Precision Medical Industry Technology Research Institute, Chongqing 400000, China; ^2^Chongqing D.A. Medical Laboratory, Chongqing 400000, China; ^3^Hangzhou D.A. Medical Laboratory, Hangzhou 310030, China; ^4^College of Laboratory Medicine, Chongqing Medical University, Chongqing 400000, China

## Abstract

**Objective:**

To identify new tumor marker genes available for early tumor screening, differentially expressed gene profiles of multiple tumors were compared using Genotype-Tissue Expression (GTEx), Cancer Cell Line Encyclopedia (CCLE), and The Cancer Genome Atlas (TCGA) databases. As AP1M2 was highly and differentially expressed in invasive breast carcinoma, the purpose of this study was to explore the association of AP1M2 gene with the survival, immune invasion, and tumor neoantigens of patients on a pan-cancer basis.

**Methods:**

The expression and distribution of AP1M2 gene in tumor tissues and the corresponding normal control tissues were analyzed using the pan-cancer databases GTEx, CCLE, and TCGA. Kaplan-Meyer survival plots and proportional hazards model (COX) were employed to evaluate actions of AP1M2 on the clinical prognosis of tumor patients. Subsequently, the association of AP1M2 expression with immune invasion in different tumor types was explored. Simultaneously, the investigation of the interrelationship of AP1M2 and tumor neoantigens of the immune system, unstable microsatellite, DNA repair genes, and DNA methyltransferases were explored, and the mutation frequency of AP1M2 gene in diverse tumors was studied. Several tumor types were analyzed using gene-set enrichment analysis (GSEA).

**Results:**

AP1M2 was abundantly expressed in a wide range of cancers, and its expression level was positively correlated with the outcome of tumor victims. Through a study on AP1M2 action on clinical prognosis and immune infiltration in tumor patients, AP1M2 expression in breast-infiltrating carcinoma was found to be highly associated with patients' overall survival and infiltration levels of macrophages, dendritic cells, T cells (CD4+ and CD8+), and B cells. Also, AP1M2 expression was positively correlated with tumor immune neoantigens and microsatellite instability in breast invasive carcinoma. The effect of AP1M2 on tumors was analyzed by GSEA, and findings demonstrated that AP1M2 expression levels in most tumors influenced the activation of tumor-associated pathways and immune-associated pathways.

**Conclusions:**

These findings suggest that AP1M2 expression levels are significantly correlated to patients' outcomes and levels of immune infiltration in most cancer types, including T cells (CD8+ and CD4+), macrophages, neutrophils, and dendritic cells (DCs), particularly in breast cancer. The results indicate that AP1M2 may influence the tumor environment of invasive breast cancer patients and it may be a target contributing to early screening and treatment for breast cancer, helping improve the efficiency of early screening and overall survival rate in invasive breast cancer patients.

## 1. Introduction

AP1M2 belongs to the adhesive protein-associated adaptor protein complex 1 that functions in the anti-Golgi network (TGN) and protein sorting in the endothelium. The adaptor-related protein complex has been characterized by mediating the recruitment of adhesive proteins to membranes and the recognition of sorting signals within the cytoplasmic tail of transmembrane cargo molecules. AP1M2 is phylogenetically conserved and expressed in all cell types detected, from yeast to mammals. Meanwhile, it is homologous in a variety of eukaryotes [1].

As little research has been made on the correlation between AP1M2 expression levels and tumorigenesis development, this study initiated a pan-cancer analysis of AP1M2 using databases TCGA, GTEx, and CCLE. Several influencing factors such as gene expression, survival status, genetic alterations, immune infiltration, and associated cellular pathways were analyzed. Meanwhile, the role of AP1M2, possible molecular mechanisms of AP1M2 in different tumor pathogeneses, and clinical outcomes were simultaneously investigated. We found that AP1M2 expression could affect survival prognosis, immune infiltration, and tumor load, as well as methylation in tumors, especially BRCA.

This research currently revealed that AP1M2 expression level in BRCA was positively associated with genetic differences, immune system, DNA methyltransferase, tumor mutational load, and microsatellite instability. This gene has the potential to be a promising target for early screening and even BRCA treatment, which can benefit patients with efficient early screening of invasive breast cancer with improved overall survival.

## 2. Materials and Methods

### 2.1. Transcriptional Data Acquisition

First, we detected expression levels of genes in 31 tissues using the GTEx dataset (https://commonfund.nih.gov/GTEx/), and further, we analyzed the gene expression levels in 31 tissues from the CCLE database (https://portals.broad.institute.org/ccle/), which was downloaded for each tumor cell line and the expression levels of 21 tissues were determined following tissue origin. mRNA data in 31 tumor samples were then obtained from the TCGA database (https://www.cancer.gov/about-nci/organization/ccg/research/structural-genomics/tcga) [2]. Data were ultimately obtained, and differences were compared by Kruskal–Wallis tests.

### 2.2. Differential Gene Expression Analysis

Subsequently, differences of AP1M2 gene expression in the tumor samples and the corresponding normal control tissues were to be determined. We downloaded TCGA Pan-Cancer and GTEx datasets from the UCSC Xena database (https://xena.ucsc.edu/). We obtained the expression difference of AP1M2 from the TCGA database of both tumor tissues and corresponding normal control tissues in 20 tumor samples. Considering limited normal tissue samples in TCGA, we synthesized normal tissue data from GTEx database and TCGA tumor tissues to determine expression differences of 27 tumors. The significance of the difference at threshold *P* < 0.05 was calculated using RStudio version 1.1.456 (RStudio Inc, USA).

### 2.3. Survival Analysis at a Pan-Cancer Level

This work assessed interrelationship between AP1M2 expression level and 33 tumor prognoses in the TCGA cohort, and univariate COX regression analyses for disease-free interval (DFI), overall survival (OS), progression-free interval (PFI), and disease-specific survival (DSS) were conducted taking into account the possible presence of nontumor mortality factors during follow-up. The threshold of Cox was *P* < 0.05. A summary forest plot was generated utilizing R package forest plot [3]. Tumors with significant correlations in the regression analysis were selected, and the samples were grouped into two at high or low expressions, referring to a median AP1M2 expression level. Hypothesis testing was performed using a log-rank test, and *P* < 0.05 was used as a threshold to calculate significant differences in survival. In addition, a correlation assessment was carried out between AP1M2 expression levels and TNM stages.

### 2.4. Relationship between AP1M2 Expression Levels and Immunity

The existence of tumor-infiltrating lymphocytes in the tumor microenvironment correlates with the improvement of outcomes and therapeutic results for different types of cancer [4]. Further investigation on whether AP1M2 expression in diverse tumors would interact with immune infiltration. It was, therefore, that we employed CIBERSORT of R package to calculate the relative proportional relationship of immunocytes in multiple tumors [5]. Their levels of immune infiltration were assessed using ESTIMATE of R package, including the immune and stromal scores of 33 tumor cell samples in the tumor microenvironment in the TCGA cohort [6]. The association between AP1M2 and the previously described indicators was analyzed using Spearman correlation analysis.

A total of 47 immune checkpoint genes were collected, and their association of expression with AP1M2 gene expression was analyzed using Spearman correlation analysis. The correlation heatmap was then created employing the R package heatmap.

### 2.5. Relationship between AP1M2 and Immune Neoantigens, Tumor Mutational Burden (TMB), and Microsatellite Instability (MSI)

Tumor neoantigens can be recognized by specific cells and encoded by mutated genes. They are mostly generated by new abnormal proteins, such as point mutation, deletion mutation, and gene fusion, and vary from those in normal cells. These proteins are enzymatically cleaved into peptide fragments and presented via DC cells to T cells as antigens. In this process, T cells can be induced to mature and activate, and characterized by tumor neoantigen-specific, thereby enabling the activated T cells to proliferate [7]. Herein, the number of neoantigens contained in tumor samples was calculated, and the results were analyzed to investigate whether there exists a correlation between AP1M2 expression levels and immune neoantigens using a Spearman correlation method [8].

TMB refers to the total number of detected somatic mutations (nonsynonymous mutations) occurring in an average 1 Mb base in the coding or exon region of malignant cell genome. It is also briefly expressed as entire nonsynonymous mutations. Meanwhile, the types of TMB mutations usually consist of single nucleotide variants (SNVs) and small insertions or deletions (Indel) [9]. In this research, the estimates of TMB in an individual tumor sample were presented separately. Spearman's rank correlation coefficient was ultimately adopted to analyze the interrelationship of the AP1M2 expression level and TMB.

MSI represents Indel of a repeat unit in malignancies, resulting in somatic alteration in the microsatellite length when compared to normal tissues. Emerging microsatellite alleles represent one phenomenon of heredity or biological inheritance [10]. PreMSIm, an R package, was utilized to predict MSI following gene expression profiles of 33 cancers, and the interrelation analysis of both gene expressions and MSI was analyzed utilizing Spearman rank correlation coefficient [11].

### 2.6. Mutation Patterns of AP1M2 in TCGA Database

The mutation details of the previously described 33 malignant tumors were downloaded from TCGA. All changes that AP1M2 developed in the tumor specimens were analyzed subsequently. Maftools, an R package, was subsequently utilized to visualize the tumors with the most AP1M2 mutations [12].

### 2.7. Correlation of AP1M2 Expression Levels with DNA Methyltransferases (DNMT) and Mismatch Repair (MMR) Genes

MMR is a mechanism of mismatch repair occurred intracellularly; the function depletion of key genes leads to irreparable DNA replication mistakes, which in turn results in higher somatic mutations. Therefore, whether AP1M2 could influence five MMR genes (MLH1, MSH2, MSH6, PMS2, and EPCAM mutations) was assessed using TCGA expression profiles.

DNA methylation also represents a mechanism that regulates relevant gene expression free from changing DNA sequences. This action mechanism enables to control of expressions of genes, resulting in chromatin structure alternation, changes in DNA conformation, and DNA stability, as well as interactions of DNA with proteins. DNA methylation is catalyzed by the action of DNA methyltransferases, and methyl groups can be added at 5′ carbon position of the cytosine ring. Thus, this study elucidated the correlation of expressions between genes and four methyltransferases (DNMT1, DNMT2, DNMT3A, and DNMT3B).

### 2.8. GSEA Analysis of Patients with Pancytopenia in TCGA

To further clarify whether AP1M2 gene expression influences tumors and in light of gene expression levels, we divided the samples into two experimental groups: a high expression group and a low expression group. KEGG enrichment analysis and signature pathways were performed in both groups using GSEA [13]. The enrichment and signature pathways of KEGG analysis were subsequently analyzed for both of the experimental groups. The c5 curated signatures were collected from the MSigDB database (https://www.gsea-msigdb.org/gsea/msigdb/collections.jsp) [9]. KEGG and HALLMARK terms and conditions were concomitantly defined in both high and low AP1M2 expression groups. FDR <0.05 was utilized to determine the significance of pathway enrichment results.

## 3. Results

### 3.1. Gene Expression Analysis Data

**Figure 1:**
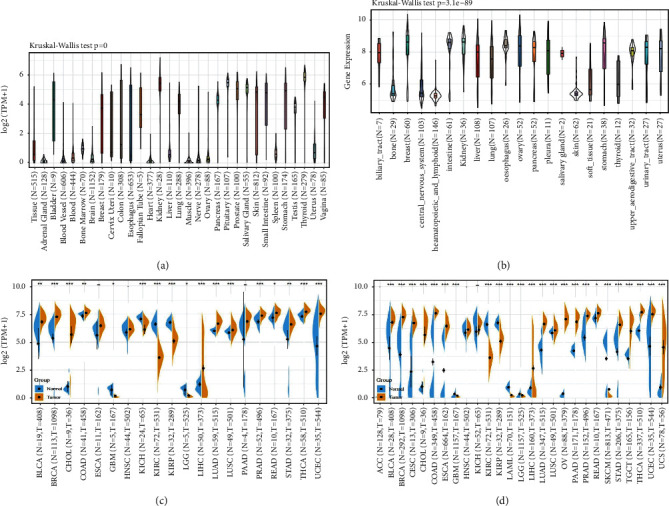
AP1M2 expression level in 31 normal tissues across (a) GTEx dataset and (b) CCLE database. The differences in gene expression between cancer and paracancerous in individual tumor samples obtained from the (c) TCGA database and (d) GTEx datasets.

We analyzed differences in gene expression between cancer and paracancer in each cancer sample obtained from the TCGA database (Figure 1(c)) in READ (*P* < 0.05), BLCA, COAD (*P* < 0.01), BRCA, CHOL, LIHC, LUAD, LUSC, PRAD, STAD (*P* < 0.001), THCA, and UCEC (*P* < 0.001), while AP1M2 expression levels in GBM, LGG (*P* < 0.05), KICH, KIRC, and KIRP (*P* < 0.001) were elevated compared with those in the normal control group (control tissues). The levels were lower than those of the relevant control normal tissues.

Evaluation of AP1M2 expression differences in LAML, OV, ACC, CESC, TGCT, and UCS was conducted after normal tissues from the GTEx dataset were set as control.  Figure 1(d) indicated highly expressed AP1M2 in CESC, OV, TGCT, and UCS (*P* < 0.001) compared with the tissues of the relevant control normal group, whereas AP1M2 was poorly expressed in LAML (*P* < 0.001) compared with those of the relevant control normal group. Unluckily, we failed to discover any significant differences in AP1M2 expression levels between ACC and its control normal.

Furthermore, the Kruskal–Wallis test showed significant differences in AP1M2 expression levels between organs (Figures 1(a) and 1(b)).

### 3.2. Survival Analysis Data

**Figure 2:**
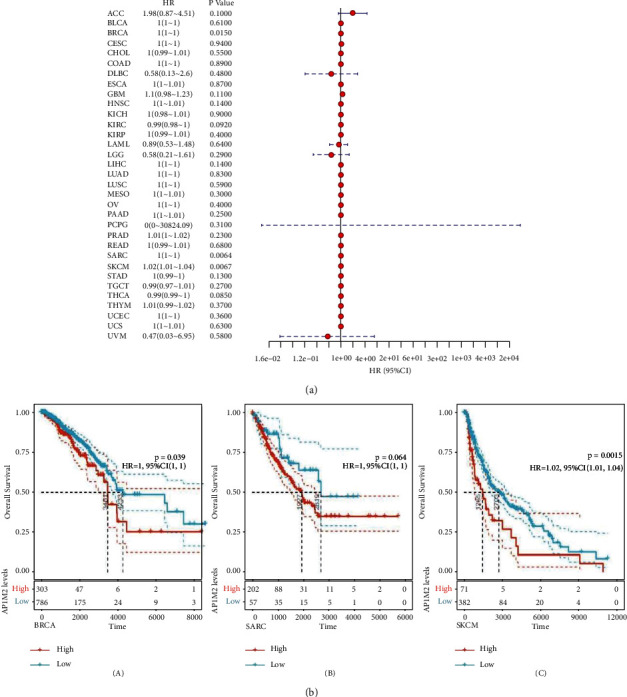
The relationship between expression and OS (overall survival time in days) in 33 tumors of TCGA. (a) The results of univariate COX regression analysis was presented via forest plot. (b) Alog-rank test was used to calculate the significance of survival differences with a threshold of *P* < 0.05, and the results were presented via Kaplan–Meier survival curves comparing survival rates of low and high expressions of AP1M2 in tumors.

We investigated the interrelationship of AP1M2 expression levels and the survival prognosis in several types of tumor patients. The association of expression levels with prognostic OS (overall survival time in days) in 33 tumors from TCGA was identified using gene expression profile data, one-way survival analysis, and forest plots in 33 tumors as shown in Figure 2(a). Meanwhile, significant tumors BRCA (*P*=0.015), SARC (*P*=0.0064), and SKCM (*P*=0.0067) were selected for prognostic KM curves. Following the expression levels of AP1M2, cancer cases were categorized into high and low expression groups, between which their correlation between AP1M2 expression and patient prognosis of different cancer types was studied using databases TCGA and GEO. As presented in Figure 2(b), highly expressed AP1M2 linked to poorer prognosis in BRCA (*P*=0.039, HR = 1, 95% CI = 1) and SKCM (*P*=0.0015, HR = 1.02, 95% CI = 1.01–1.04).

**Figure 3:**
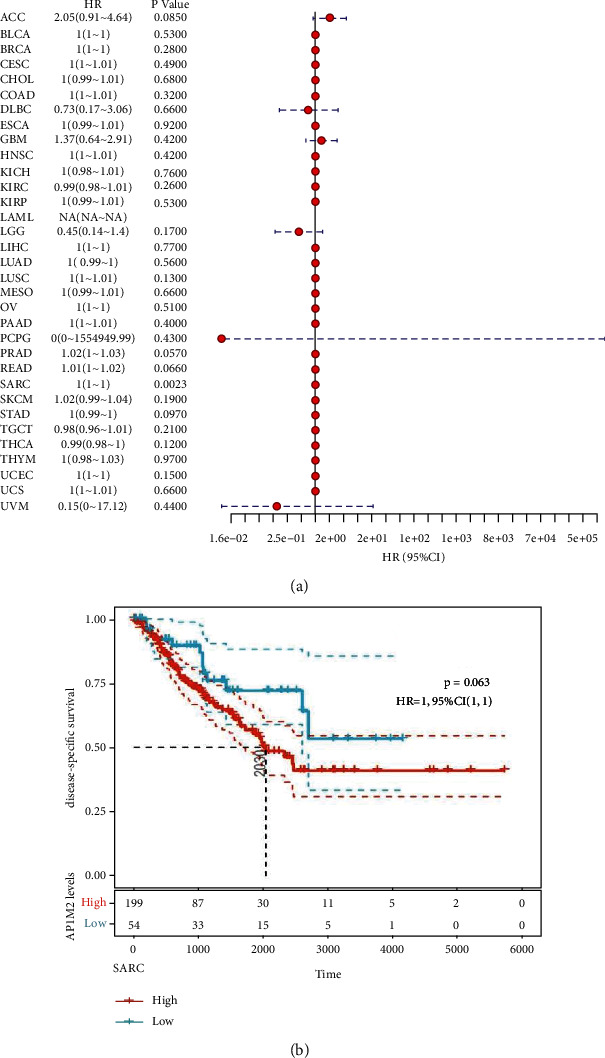
The relationship between expression and DFS (disease-specific survival) in 33 tumors of TCGA. (a) The results of univariate COX regression analysis were presented via forest plot. (b) A log-rank test was used to calculate the significance of survival differences with a threshold of *P* < 0.05, and the results were presented via Kaplan–Meier survival curves comparing the survival rates of low and high expression of AP1M2.

Meanwhile, considering the presence of nontumor death factor during the follow-up period, the correlation between the gene expression of 33 tumors and the prognostic DSS in TCGA was initially analyzed (Figure 3(a)). The significant tumor SARC (*P*=0.0023) was selected according to the expression level of AP1M2, and the cancer samples were grouped into high and low expression experimental groups for prognostic KM curves. The results failed to reveal any positive correlated features between AP1M2 expression and DSS in SARC (Figure 3(b)) (*P*=0.063, HR = 1, 95% CI = 1).

**Figure 4:**
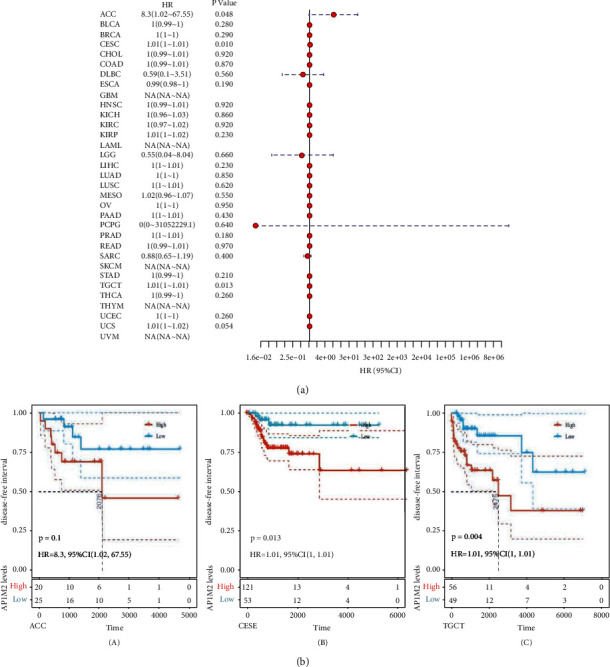
The relationship between expression and DFI (disease-free interval) in 33 tumors of TCGA. (a) The results of univariate COX regression analysis were presented via forest plot. (b) A log-rank test was used to calculate the significance of survival differences with a threshold of *P* < 0.05, and the results were presented via Kaplan–Meier survival curves comparing the survival rates of low and high expression of AP1M2 in tumors.

**Figure 5:**
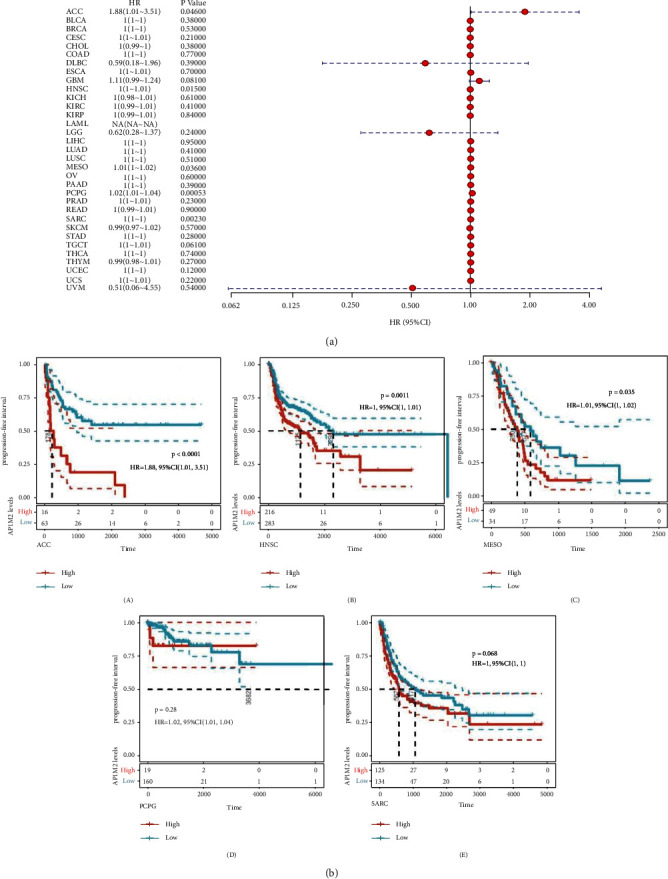
The relationship between expression and PFI (progression-free interval) in 33 tumors of TCGA. (a) The results of univariate COX regression analysis were presented via forest plot. (b) A log-rank test was used to calculate the significance of survival differences with a threshold of *P* < 0.05, and the results were presented via Kaplan–Meier survival curves comparing the survival rates of low and high expression of AP1M2 in tumors.

Next, the same procedures were carried out to explore whether there existed correlations between gene expressions and prognostic DFI (Figure 4(a)) and PFI (Figure 5(a)) in 33 tumors of TCGA. There were significant correlation in ACC (*P*=0.048), CESC (*P*=0.01), TGCT (*P*=0.013) and ACC (*P*=0.046), HNSC (*P*=0.015), MESO (*P*=0.036), PCPG (*P*=0.00053), and SARC (*P*=0.0023), and two high and low expression groups were divided in light of the AP1M2 levels for prognostic KM curves. As shown in Figure 4(b), the DFI survival analysis revealed that there was an association of higher AP1M2 expression with poorer prognosis in CESC (*P*=0.013, HR = 1.01, 95% CI = 1–1.01) and TGCT (*P*=0.004, HR = 0.01, 95% CI = 1–1.01). As shown in Figure 5(b), the same propensity of PFI survival analysis was revealed as that of the previously described conditions that highly expressed AP1M2 corresponded to poorer prognosis in ACC (*P* < 0.0001, HR = 1.88, 95% CI = 1.01–3.51), HNSC (*P*=0.0011, HR = 1, 95% CI = 1–1.01), and MESO (*P*=0.035, HR = 1.01, 95% CI = 1–1.02).

### 3.3. Association of Gene Expression with Immunity Infiltration Levels

**Figure 6:**
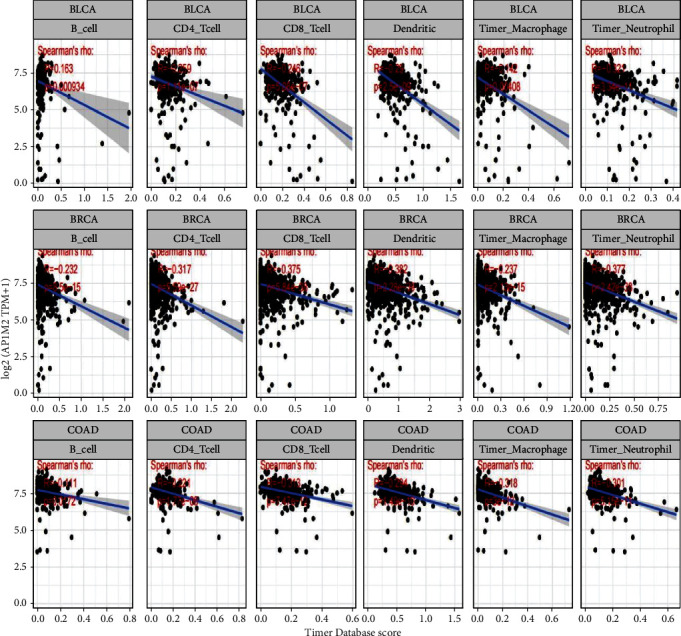
We used the CIBERSORT method in the R package to calculate the relative proportional relationship of immune cells in multiple tumors. The three most significantly correlated tumors BLCA, BRCA, and COAD were selected.

Tumor-infiltrating lymphocytes consist of cells invading cancer tissues, and they function as independent biomarkers for the prediction of anterior lymph node status and efficacy of cancer treatment [14]. We investigated whether this gene expression linked to immune invasion in different cancer types and figured out that AP1M2 expression levels were positively correlated with the levels of B-cell infiltration in 14 cancers, CD4+ T cell infiltration in 17 cancers, CD8+ T cells in 16 cancers, macrophages in 19 cancers, neutrophils in 19 cancers, and dendritic cells in 19 cancers. The three most significantly correlated tumors BLCA, BRCA, and COAD were selected (Figure 6). AP1M2 expression levels in BLCA, BRCA, and COAD (all *P* < 0.0001) were all significantly and negatively related to B cells, T cells (CD4+ and CD8+), macrophages, neutrophils, and DC.

**Figure 7:**
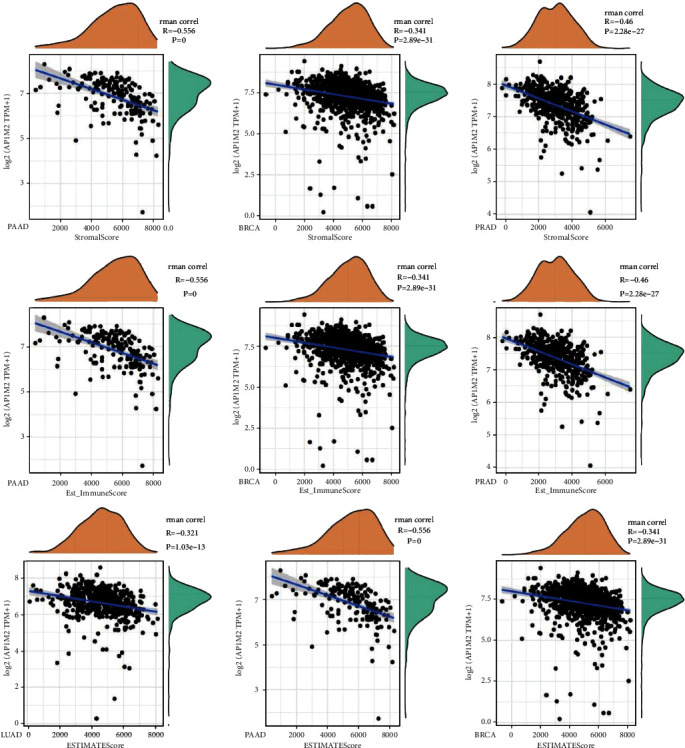
Correlation of AP1M2 expression with the immune score, ESTIMATE score, and stromal score in PAAD, BRCA, PRAD, and LUAD.

Numerous researchers have demonstrated that the tumor immune microenvironment determines the occurrence and development of a wide variety of tumors [15]. Following the visualization of the interrelationships between gene expression and scores of the immune system, stromal, and ESTIMATE in the 33 reported tumors, we selected three tumors with the most significant relationship in each score (Figure 7). AP1M2 levels were more significant in PAAD (RS = −0.556, PS < 0.0001, RI = -0.517, PI < 0.001) and BRCA (RS = −0.341, PS = 2.89*e* − 31, RI = −0.385, PI = 3.24*e* − 40). PRAD (RS = −0.46, PS = 2.28*e* − 27, RI = −0.401, PI = 1.24*e* − 20) was negatively correlated between the expression levels in PRAD (RS = −0.46, PS = 2.28*e* − 27, RI = −0.401, PI = 1.24*e* − 20) and the stromal and immune scores. However, as for LUAD (*R* = −0.374, *P* < 0.0001), PAAD (*R* = −0.564, *P* < 0.0001), and BRCA (*R* = −0.409, *P*=1.41*e* − 45), AP1M2 gene expression level had a negative correlation with composite scores.

**Figure 8:**
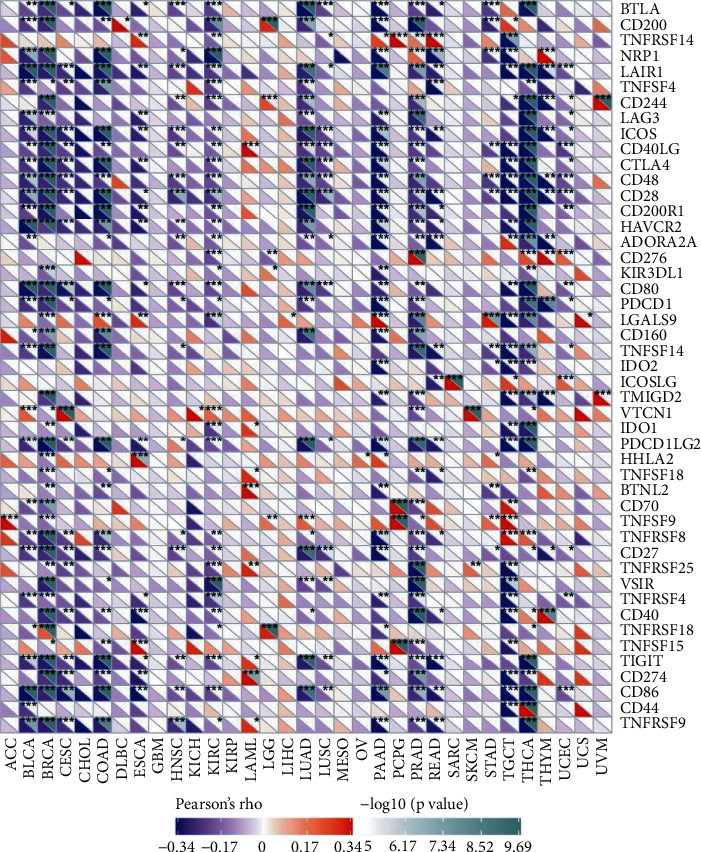
The relationship between AP1M2 and the expression of immune checkpoint genes is presented via heatmap. The horizontal coordinate indicates the 33 selected tumors, and the vertical coordinate indicates the relevant immune checkpoints, where ^*∗*^indicates correlation (*P* < 0.05), ^*∗∗*^indicates high correlation (*P* < 0.01), and ^*∗∗∗*^indicates significant correlation (*P* < 0.001).

Under normal conditions, immune cells can recognize tumor cells and remove them from the tumor microenvironment [16]. Tumor immunotherapy has been recognized in medicine by reactivating and maintaining the tumor immune cycle to suppress and eliminate immune cells as a way to repair the body's normal antitumor immune response. The current widely applied immune checkpoints are inhibitors of monoclonal antibody-based immune checkpoints and small molecules, antibody therapeutics, and cancer treatment vaccines, as well as cytotherapy [17]. As shown in Figure 8, the horizontal coordinate indicates the 33 selected tumors and the vertical coordinate indicates the relevant immune checkpoints, where ^*∗*^indicates correlation (*P* < 0.05), ^*∗∗*^indicates high correlation (*P* < 0.01), and ^*∗∗∗*^indicates significant correlation (*P* < 0.001). Higher AP1M2 expression indicated poorer prognosis of tumor patients by the survival analysis of BRCA, while AP1M2 expression levels were negatively correlated with B cells, T cells (CD4+ and CD8+), macrophages, neutrophils, DC infiltration, and scores of the immune system, stromal, and composites via immune analysis. These results suggested a specific role of AP1M2 in the prognostic analysis and immune infiltration in BRCA. According to Figure 6, the immune checkpoint genes were positively associated with BRCA immunity including BTLA, CD200, NRP1, LAIR1, TNFSF4, CD244, LAG3, ICOS, CD40LG, CTLA4, CD48, CD28, CD200R1, HAVCR2, CD80, LGALS9, CD160, TNFSF14, TMIGD2, PDCD1LG2, HHLA2, TNFSF18, CD70, TNFSF9, TNFRSF8, CD27, VSIR, TNFRSF4, CD40, TNFRSF18, TIGIT, CD274, CD86, and TNFRSF9.

### 3.4. Relationship between Gene Expression and Immune Neoantigens, TMB, and MSI

**Figure 9:**
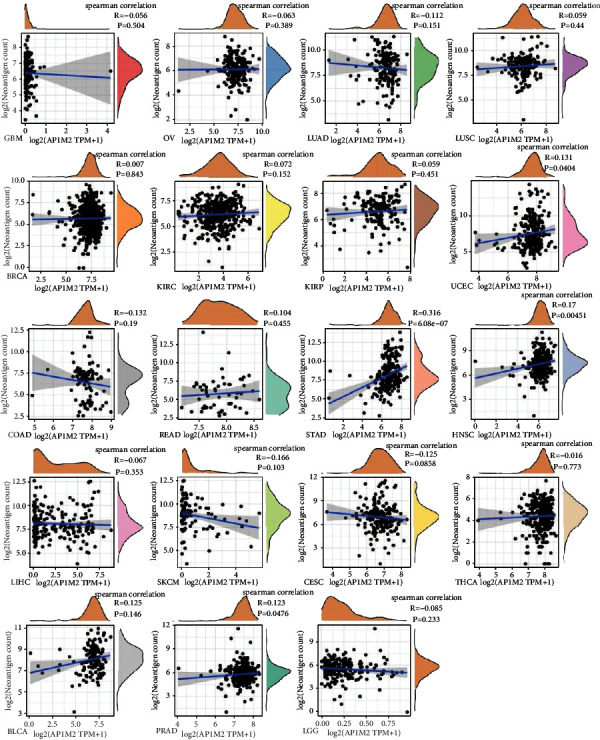
Correlation of AP1M2 expression with neoantigens.

Neoantigen vaccines can be designed and synthesized using strong immunogenicity and heterogeneity of tumor neoantigens according to the tumor cell mutations, which will benefit patients with a satisfactory therapeutic effect after immunization [18]. By counting the neoantigen quantity of every sample tumor, we subsequently studied whether there was any association with AP1M2 expression. As shown in Figure 9, the expression levels of AP1M2 in UCEC (*R* = 0.131, *P* < 0.0404), PRAD (*R* = 0.123, *P* < 0.0476), HNSC (*R* = 0.17, *P* < 0.0045), and STAD (*R* = 0.316, *P*=6.08*e* − 07) showed a link to the number of neoantigens.

**Figure 10:**
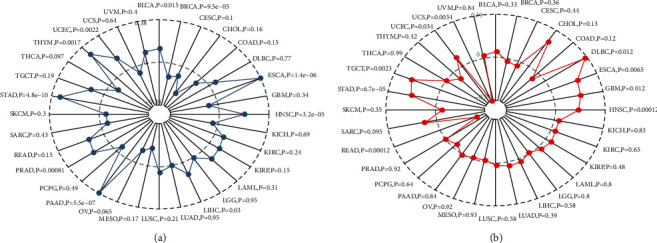
Correlation between AP1M2 and TMB (a) and microsatellite instability (b).

As TMB predicts favorable responses to immune checkpoint inhibitors, mutated cell count can be calculated from the tumors applying Spearman rank correlation coefficient. Meanwhile, its association with gene expression was also analyzed, as shown in Figure 10(a). The results were as follows: the AP1M2 gene expression level in BRCA (*P*=9.5*e* − 05) was significantly and negatively correlated with TMB, whereas those expression levels revealed in BLCA (*P*=0.015), ESCA (*P*=1.4*e* − 06), HNSC (*P*=3.2*e* − 05), LIHC (*P*=0.03), PAAD (*P*=5.5*e* − 07), STAD (*P*=4.8*e* − 10), THYM (*P*=0.0017), and UCEC (0.0022) were positively correlated with TMB. Among them, AP1M2 levels in STAD, PAAD, and ESCA were most significantly related to TMB.

Whether gene expression and MSI had a connection was subsequently verified applying Spearman rank correlation coefficient (Figure 10(b)). The results indicated that the AP1M2 gene expression levels in DLBC (*P*=0.012), ESCA (*P*=0.0065), GBM (*P*=0.012), HNSC (*P*=0.00012), STAD (*P*=6.7*e* − 05), and TGCT (*P*=0.0023) were positively correlated with MSI, whereas those in the UCS (*P*=0.0034) and READ (*P*=0.00012) were negatively correlated with MSI.

### 3.5. Mutation Patterns of Genes in TCGA Tumor Samples

**Figure 11:**
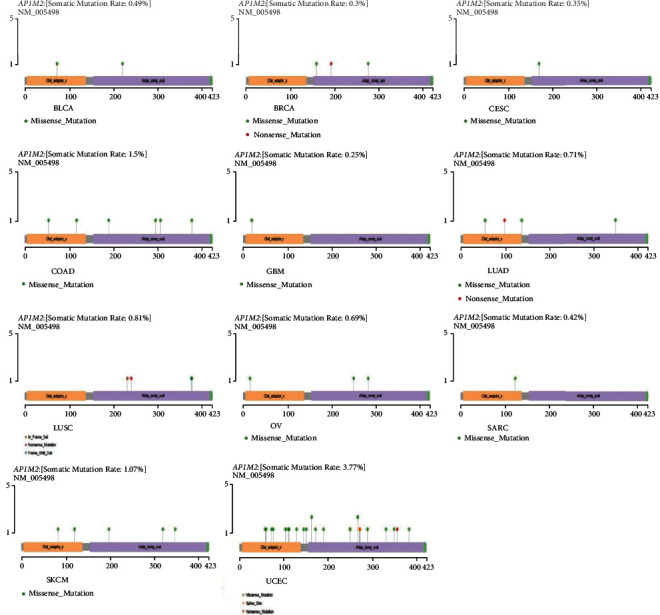
AP1M2 gene mutation patterns in several tumors.

Mutated AP1M2 was further analyzed following data collection of the 33 tumors through the TCGA database. As shown in Figure 11, mutations of AP1M2 only occurred in BLCA, BRCA, CESC, COAD, GBM, LUAD, LUSC, OV, SARC, SKCM, and UCEC after observation. Among them, the top three tumors with the highest AP1M2 mutation rates were UCEC (3.77%), COAD (1.5%), and SKCM (1.07%), indicating that AP1M2 was rarely mutated in most tumors.

### 3.6. Gene Expression TCGA Tumor Samples concerning DNA MMR and Methyltransferases

**Figure 12:**
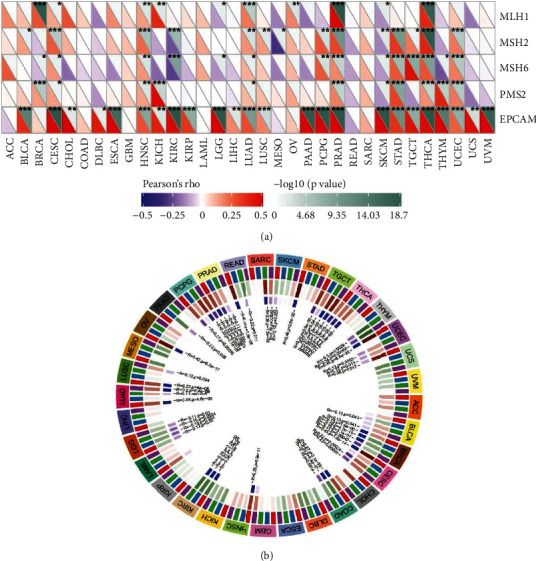
The relationship between AP1M2 expression level and (a) mismatch repair genes and (b) DNA methyltransferase. ^*∗*^*P* < 0.05, ^*∗*^*P* < 0.01; ^*∗∗∗*^*P* < 0.001.

In light of the TCGA expression profile data, we subsequently assessed the interrelationship between mutations of the five MMR genes MLH1, MSH2, MSH6, PMS2, and EPCAM and gene expressions (Figure 12(a)). The results revealed that AP1M2 expression levels were significantly correlated with the five MMR genes in CESC, HNSC, LUAD, PRAD, SKCM, and THCA.

We simultaneously analyzed the visualization of expression correlation between AP1M2 and the previously described four methyltransferases (DNMT1: red, DNMT2: blue, DNMT3A: green, and DNMT3B: purple) (Figure 12(b)). The results indicated that expression levels of UCEC, BRCA, CESC, COAD, KIRC, LGG, LUAD, PRAD, TGCT, THCA, and PCPG, AP1M2 were substantially correlated with the four genes, with AP1M2 expression levels in TGCT (*R* = 0.38, *P*=3.3*e* − 06) being the most significantly correlated.

### 3.7. GSEA Analysis

**Figure 13:**
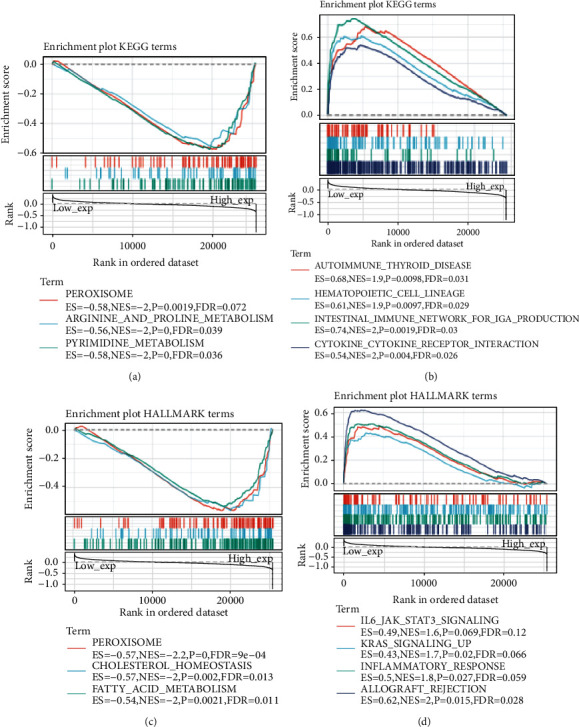
The result of GSEA.

We employed two groups of tumor specimens to verify actions of gene expression on tumors: a high and a low expression groups in accordance with gene expression. GSEA was employed for KEGG enrichment and HALLMARK pathway analysis in both expression groups. Subsequently, three pathways were selected, which presented the most significant GSEA results (Figure 13). KEGG pathway analysis in Figure 13 exhibited that high expression of AP1M2 mainly activated PEROXISOME (ES = −0.58, NES = −2, *P*=0.0019, FDR = 0.072), ARGININE_AND_PROLINE_METABOLISM (ES = −0.56, NES = −2, *P* < 0.001, FDR = 0.039), and PYRIMIDINE_METABOLISM (ES = −0.58, NES = −2, *P* < 0.001, FDR = 0.036), while poorly expressed AP1M2 mainly activated AUTOIMMUNE_THYROID_DISEASE (ES = 0.68, NES = 1.9, *P*=0.0098, FDR = 0.031), HEMATOPOIETIC_CELL_LINEAGE (ES = 0.61, NES = 1.9, *P*=0.0097, FDR = 0.029), INTESTINAL_IMMUNE_NETWORK_FOR_IGA_PRODUCTION (ES = 0.74, NES = 2, *P*=0.0019, FDR = 0.03), and CYTOKINE_CYTOKINE_RECEPTOR_INTERACTION (ES = 0.54, NES = 2, *P*=0.004, FDR = 0.026). In the HALLMARK pathway, the highly expressed AP1M2 mainly activated PEROXISOME (ES = −0.57, NES = −2.2, *P*=0, FDR = 9*e* − 04), CHOLESTEROL_HOMEOSTASIS (ES = −0.57, NES = −2, *P*=0.002, FDR = 0.013), and FATTY_ACID_METABOLISM (ES = −0.54, NES = −2, *P*=0.0021, FDR = 0.011), whereas poorly expressed AP1M2 mainly activated IL6_JAK_STAT3_SIGNALING (ES = 0.49, NES = 1.6, *P*=0.069, FDR = 0.12), KRAS_SIGNALING_UP (ES = 0.43, NES = 1.7, *P*=0.02, FDR = 0.066), INFLAMMATORY_RESPONS (ES = 0.5, NES = 1.8, *P*=0.027, FDR = 0.059), and ALLOGRAFT_REJECTION (ES = 0.62, NES = 2, *P*=0.015, FDR = 0.028).

## 4. Discussion

Being one of the most densely populated countries, China has achieved remarkable progress in the improvement of people's health over the last several decades. As the population ages, China's burden of cancer expenses keeps growing [19]. Meanwhile, since the outbreak of the novel coronavirus pandemic in 2019, several studies have demonstrated that individuals under a high risk of COVID-19 include cancer patients who are immunosuppressed throughout the body [20].

Through literature retrieval, little literature on AP1M2 pan-cancer analysis of the overall tumor has been found. Therefore, based on data from TCGA, CCLE, UCSC Xena, and GTEx databases, as well as gene expression, gene variants, methylation, immune infiltration, and enrichment analysis, a comprehensive exploration was conducted on AP1M2 gene from the 33 different tumor types in the TCGA cohort. The findings indicated that the AP1M2 expression level exhibited a positive link to the prognosis and immune aspects of several different tumors, especially breast-infiltrating carcinomas. Hence, AP1M2 may be applied as a screening indicator and therapeutic target for multiple tumors in the future.

AP1M2 expression differences were revealed simultaneously among various cancers and normal control, which indicated that AP1M2 was highly expressed and significant in breast cancer, liver cancer, lung cancer, bile duct cancer, prostate cancer, gastric cancer, thyroid cancer, and common genital tumors compared to normal tissues. Conversely, some datasets also showed that AP1M2 was poorly expressed in kidney cancer and acute myeloid leukemia compared to normal tissues in the control group. AP1M2, also known as Mu-2, has been shown that Mu-2-related death-inducing gene (MuD) is a 490-amino-acid protein belonging to the medium subunit family of adaptin protein (AP), which can independently induce cancer cell death in association with adhesive protein-mediated endocytosis found in the Mu-2 subunit of the articulation protein [21]. Therefore, AP1M2 may also possess the function of inducing cancer cell death. However, such speculation is inconsistent with gene expression analysis and survival analysis, and more investigations remain indispensable to reveal actions and mechanisms of AP1M2 among a range of cancer types.

Following the analysis of AP1M2 expression levels and immunity, we found that AP1M2 expression in BRCA was negatively associated not only with B cells, CD4+ T cells, CD8+ T cells, macrophages, neutrophils, and DC (Figure 4), but also with scores of the immune system, stromal, and composite in ESTIMATE analysis (Figure 5). The occurrence and progress of a tumor are complicated, and the processes in which cancer cells interact with microenvironment and immune system influence tumorigenesis and progression [22]. Furthermore, immunocytes have a pivotal secondary role in maintaining tissue integrity and normal functions by eliminating pathogens in different states of homeostasis, infection, and noninfectious disturbances of the body and have an impact on the clinical outcome of tumors [23]. In addition, it has been shown that immune scores in RBCA at either a high or moderate level can lead to improved disease-free survival or OS [24]. Hence, the association of increased AP1M2 expression levels with poor prognosis in BRCA patients may be related to the fact that AP1M2 expression suppresses immunocyte infiltration into tumor microenvironment and decreased immune score.

In BRCA, the AP1M2 expression level was significantly and negatively related to most immune checkpoints except TNFRSF18 (Figure 6). Immune checkpoints represent multiple inhibitions and stimulation pathways for immunocytes to maintain their immunologic tolerance and adjust corresponding immune responses to dangerous physical signals [25]. Immune checkpoint blockade can either retard or suppress evasion of tumor cells and slow down tumor growth. Through inquiring literature, we found that high expression levels of CTLA-4 and TIGIT were associated with a good prognosis of BRCA [26]. Figure 6 presented that AP1M2 levels were correlated with both CTLA-4 and TIGIT.

Several investigations have revealed that TMB is critical for cancer development and progression, and cancer patients with high TMB levels responded more strongly to immunotherapy than low TMB level patients, which is also associated with cancer prognosis [27–29]. However, through the analysis of AP1M2 expression levels and TMB (Figure 7), AP1M2 expression levels in BRCA were significantly negatively correlated with TMB. Thus, increased AP1M2 expression levels may lead to lower TMB in patients, which might be less sensitive to immunotherapy.

In addition, a positive correlation was revealed between AP1M2 expression and MMR and DNA methyltransferases in BRCA. Taken together, AP1M2 may be used as a prognostic predictor or a therapeutic target of BRCA for immunotherapy in clinical settings for the improvement of patients' prognosis and survival rates. In future research, we plan to use gene editing methods to overexpress or knock out AP1M2 in tumor cells and animal models to verify the function and molecular regulation mechanism of AP1M2. Through these studies, it is expected that the clinical application potential of AP1M2 will be further explored.

## Data Availability

The data used to support the findings of this study are included within the article.
